# The Impact of the Network Topology on the Viral Prevalence: A Node-Based Approach

**DOI:** 10.1371/journal.pone.0134507

**Published:** 2015-07-29

**Authors:** Lu-Xing Yang, Moez Draief, Xiaofan Yang

**Affiliations:** 1 College of Computer Science, Chongqing University, Chongqing, China; 2 Department of Electrical and Electronic Engineering, Imperial College London, South Kensington Campus, London, United Kingdom; 3 School of Software Engineering, Chongqing University, Chongqing, China; Shanxi University, CHINA

## Abstract

This paper addresses the impact of the structure of the viral propagation network on the viral prevalence. For that purpose, a new epidemic model of computer virus, known as the node-based SLBS model, is proposed. Our analysis shows that the maximum eigenvalue of the underlying network is a key factor determining the viral prevalence. Specifically, the value range of the maximum eigenvalue is partitioned into three subintervals: viruses tend to extinction very quickly or approach extinction or persist depending on into which subinterval the maximum eigenvalue of the propagation network falls. Consequently, computer virus can be contained by adjusting the propagation network so that its maximum eigenvalue falls into the desired subinterval.

## 1 Introduction

The rapidly popularized Internet has brought us lots of benefits. On the flip side of the coin, computer viruses can propagate their replicates through the Internet much more rapidly than ever before, resulting in great disruptions. Although antivirus software is recognized as the major means of defending against electronic viruses, there is a marked lag from the appearance of a new virus to the availability of its vaccine.

As an important supplement to antivirus techniques, the epidemic dynamics of computer viruses aims to understand the laws governing the spread of malware on networks and, thereby, to work out proper strategies to contain the viral prevalence. Since Kephart and White’s seminal work on the compartment modeling of computer viruses in the early 1990s [1, 2], a multitude of compartment-based computer virus propagation models, ranging from the SIR models [3] and the SIRS models [4, 5] to the SEIRS models [6], have been suggested. Most of these models are suited to infectious diseases and computer viruses equally well. In reality, however, some computer viruses have peculiarities most infectious diseases do not have. As we know, for most infectious diseases, there is a non-ignorable interval from the time when an individual gets infected to the time when it can infect other individuals. As opposed to this, for most computer viruses, one computer can infect other computers as soon as it gets infected. To capture this common feature of most computer viruses, a series of epidemic models of computer virus, named as the SLBS models, were proposed [7, 8].

The network through which computers communicate with one another is frequently used to propagate viruses, and it has been recognized that the structure of the network has significant impact on the prevalence of virus [9]. In the early 2000s, it was empirically found that many real-world networks, ranging from the Internet and the World Wide Web to some email networks, are highly structured [10–12]. Later, a wave of research on virus epidemic dynamics was initiated, with focus on the propagation of virus on scale-free networks [13–19].

One common defect of all compartment-based epidemic models is that only partial knowledge on the network topology (the degree distribution or the degree correlation, say) can be used when establishing such models. In sharp contrast to this, when establishing a node-based epidemic model, one can make the best of the complete knowledge on the network topology [20]. As a result, some interesting properties concerning the viral spread, ranging from the mean propagation time and the expected number of infected nodes to the most probable network state, have been found [21–25].

With the aid of a node-based epidemic model, Wang et al. [26] found that whether viruses approach extinction depends heavily on the spectral radius of the underlying network. Next, by studying the *N*-interwined SIS model, Mieghem et al. [27] found that whether viruses decline toward extinction depends on the maximum eigenvalue of the network. Later, by examining a node-based SIR model, Youssef and Scoglio [28] indicated that the maximum number of infected nodes is closely related to the spectrum of the network. For more information on this topic, see Refs. [29–34].

This paper addresses the impact of the network topology on the viral prevalence, provided that a computer can infect other computers as soon as it gets infected. For that purpose, a node-based virus epidemic model, known as the *node-based SLBS model*, is proposed. After exhaustive research, it is found that the maximum eigenvalue of the underlying network is a key factor determining the viral prevalence. Specifically, the value range of the maximum eigenvalue is partitioned into three subintervals: viruses tend to extinction very quickly or approach extinction or persist depending on into which subinterval the maximum eigenvalue of the network falls. Consequently, computer virus can be contained by adjusting the network topology so that its maximum eigenvalue falls into the desired subinterval. Numerical examples support our results.

The rest of this paper is organized as follows: Preliminary knowledge is presented in Section 2, and the compartment-based SLBS models are briefly reviewed in Section 3. Section 4 describes the *node-based SLBS model*, Section 5 conducts a comprehensive analysis of this model, Section 6 gives some numerical examples, and Section 7 discusses the potential applications of the proposed model. Finally, Section 8 summarizes this work and presents some topics that are worthy of study.

## 2 Preliminaries

In this paper, the underlying network through which viruses propagate is denoted by a simple graph *G* = (*V*, *E*) on *N* non-isolated nodes numbered 1 through *N*, where nodes stand for terminal devices of the network, and edges stand for network links through which viruses can propagate. Let **A** = [*a*_*ij*_]_*N* × *N*_ denote the adjacency matrix of graph *G*, let {*d*_*k*_,1 ≤ *k* ≤ *N*} denote the degree sequence of *G*, and let {*λ*_*k*_,1 ≤ *k* ≤ *N*} denote the spectrum of **A**. As **A** is real and symmetric, we may assume *λ*_max_ = *λ*_1_ ≥ *λ*_2_ ≥ ⋯ ≥ *λ*_*N*_.

For the purpose of analyzing the new computer virus epidemic model introduced in the next section, we need the following two lemmas.

**Lemma 1** [35] *Consider a smooth dynamical system* $\frac{d\text{x}(t)}{dt}=\text{f}(\text{x}(t))$ *defined at least in a compact set* *C*. *C* *is positively invariant if for any smooth point* **y** *of* ∂*C*, **f**(**y**) *is pointing into* *C*.

**Lemma 2** [36] *Consider an* *n*-*dimensional dynamical system*

$$\begin{array}{c} \frac{dx(t)}{dt}=Bx\left(t\right)+G\left(x\left(t\right)\right), \end{array}$$

*where* **x**(0) ∈ Ω, *a positively invariant compact convex set containing the origin*, **G**(**x**) ∈ *C* ^1^(Ω), ${\text{lim}}_{\text{x}\to \text{0}}\frac{\left\|\text{G}(\text{x})\right\|}{\left\|\text{x}\right\|}=0$. *Suppose matrix* **B** ^*T*^ *has a real eigenvector* **z** *such that*

*(C1)* ${\text{inf}}_{\text{x}\in \Omega -\{\text{0}\}}\frac{\left\langle \text{x},\text{z}\right\rangle }{\left\|\text{x}\right\|}>0$,
*(C2)* sup_**x** ∈ Ω_⟨**G**(**x**), **z**⟩ ≤ 0, *and*
*(C3)* *the origin forms the largest positively invariant set included in the set* {**x** ∈ Ω∣⟨**G**(**x**), **z**⟩ = 0}.

*Let* *s*(**B**) *denote the maximum real part of all eigenvalues of* **B**. *Then, we have*

*(a)* *s*(**B**) < 0 *implies the global stability of the origin, and*
*(b)* *s*(**B**) > 0 *implies that* **x**(0) ≠ **0** ⇒ liminf_*t* → ∞_‖**x**(*t*)‖ > 0.

**Lemma 3** [37] *For a graph* *G* *with* {*d*_*k*_,1 ≤ *k* ≤ *N*} *as the degree sequence, its largest eigenvalue* *λ*_max_ *has the following bounds*.

$$\begin{array}{c} \sqrt{\frac{1}{N}\sum_{i}d_{i}^{2}}\leq \lambda_{\max}\leq \min\{\sqrt{\frac{N-1}{N}\sum_{i}d_{i}},\max_{i}d_{i}\} \end{array}$$

## 3 A brief review of the compartment-based SLBS models

This section gives a brief review of the previously proposed SLBS models.

Under an SLBS model, every node in a network is assumed to be in one of three possible states: *susecptible*, i.e. uninfected, *latent*, i.e., infected and with all virues in the node being in their latent phase, and *exploding*, i.e., infected and with at least one virus in the node being in its exploding phase. For a compartment-based SLBS model, all nodes in a network are grouped into three classes (i.e., compartments) according to their states, and the change in the fraction of each compartment is the focus of study.

The original compartment-based SLBS models were established based on the homogeneously mixed assumption of the propagation network [7, 8]. However, most real-world networks, including the world-wide-web and the Internet, have been impirically found to be highly structured rather than simply homogeneously [11]. Therefore, a new compartment-based SLBS model was later suggested based on the assumption that the propagation network admits a prescribed degree distribution [18].

All of the above mentioned SLBS models suffer from a common defect that it is not possible to make full use of the knowledge concerning the structure of the propagation network. As a result, it is extremely difficult to deeply understand the impact of the network topology on the viral prevalence by solely studying such compartment-based models.

## 4 The new computer virus epidemic model

As with the traditional compartment-based SLBS models [8, 18], at any time, each and every node in the network is in one of three possible states: *susceptible*, *latent*, and *exploding*. Let *X*_*i*_(*t*) = 0 (respectively, 1, 2) stands for that node *i* is susceptible (respectively, latent, exploding) at time *t*. Then the state of the whole network at time *t* can be represented by the vector

$$\begin{array}{c} X\left(t\right)=[X_{1}\left(t\right),X_{2}\left(t\right),...,X_{N}\left(t\right)]. \end{array}$$

Let *s*_*i*_(*t*) (respectively, *l*_*i*_(*t*), *b*_*i*_(*t*)) denote the probability of the event that node *i* is susceptible (respectively, latent, exploding) at time *t*,

$$\begin{array}{c} s_{i}\left(t\right)=\mathrm{Pr}\left(X_{i}\left(t\right)=0\right),\;l_{i}\left(t\right)=\mathrm{Pr}\left(X_{i}\left(t\right)=1\right),\;b_{i}\left(t\right)=\mathrm{Pr}\left(X_{i}\left(t\right)=2\right). \end{array}$$

Now, let us impose a set of statistical assumptions on the state transitions of a node.

(H1) A susceptible node is infected by a latent (respectively, exploding) neighbor with probability per unit time *β*_1_ (respectively, *β*_2_). As a result, when the number of infected nodes is small, a susceptible node *i* gets infected approximately with average probability per unit time *β*_1_∑_*j*_ *a*_*ij*_ *l*_*j*_(*t*)+*β*_2_∑_*j*_ *a*_*ij*_ *b*_*j*_(*t*). As the mission of all the viruses staying in a latent node is to infect other nodes, whereas the mission of all the exploding viruses staying in an exploding node is to destruct the system, we assume *β*_1_ > *β*_2_.
(H2) Some virus in a latent node breaks out with probability per unit time *α*.
(H3) A latent (respectively, exploding) node gets cured with probability per unit time *γ*_1_ (respectively *γ*_2_). As an exploding node has more chance to be cured than a latent node, we assume *γ*_2_ > *γ*_1_.

**Fig 1:**
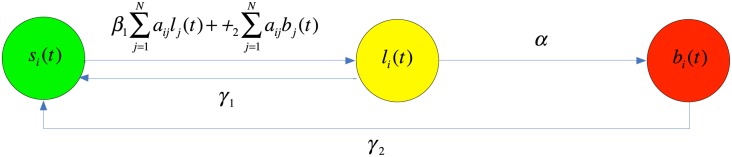
Diagram of assumptions (H1)-(H3).

Fig 1 shows these assumptions schematically.

Let Δ*t* be a very small time interval. By the total probability formula, we have the following relations:

$$\begin{array}{ccc} s_{i}\left(t+\Delta t\right) & = & s_{i}\left(t\right)\mathrm{Pr}\left(X_{i}\left(t+\Delta t\right)=0|X_{i}\left(t\right)=0\right)+l_{i}\left(t\right)\mathrm{Pr}\left(X_{i}\left(t+\Delta t\right)=0|X_{i}\left(t\right)=1\right)\\ & & +b_{i}\left(t\right)\mathrm{Pr}\left(X_{i}\left(t+\Delta t\right)=0|X_{i}\left(t\right)=2\right),\\ l_{i}\left(t+\Delta t\right) & = & s_{i}\left(t\right)\mathrm{Pr}\left(X_{i}\left(t+\Delta t\right)=1|X_{i}\left(t\right)=0\right)+l_{i}\left(t\right)\mathrm{Pr}\left(X_{i}\left(t+\Delta t\right)=1|X_{i}\left(t\right)=1\right)\\ & & +b_{i}\left(t\right)\mathrm{Pr}\left(X_{i}\left(t+\Delta t\right)=1|X_{i}\left(t\right)=2\right),\\ b_{i}\left(t+\Delta t\right) & = & s_{i}\left(t\right)\mathrm{Pr}\left(X_{i}\left(t+\Delta t\right)=2|X_{i}\left(t\right)=0\right)+l_{i}\left(t\right)\mathrm{Pr}\left(X_{i}\left(t+\Delta t\right)=2|X_{i}\left(t\right)=1\right)\\ & & +b_{i}\left(t\right)\mathrm{Pr}\left(X_{i}\left(t+\Delta t\right)=2|X_{i}\left(t\right)=2\right). \end{array}$$

Assumptions (H1)-(H3) imply the following equations:

$$\begin{array}{ccc} \mathrm{Pr}(X_{i}\left(t+\Delta t\right)=0|X_{i}\left(t\right)=0) & = & 1-[\beta_{1}\sum_{j}a_{ij}l_{j}\left(t\right)+\beta_{2}\sum_{j}a_{ij}b_{j}\left(t\right)]\Delta t+o\left(\Delta t\right),\\ \mathrm{Pr}(X_{i}\left(t+\Delta t\right)=1|X_{i}\left(t\right)=0) & = & [\beta_{1}\sum_{j}a_{ij}l_{j}\left(t\right)+\beta_{2}\sum_{j}a_{ij}b_{j}\left(t\right)]\Delta t+o\left(\Delta t\right),\\ \mathrm{Pr}(X_{i}\left(t+\Delta t\right)=2|X_{i}\left(t\right)=0) & = & o(\Delta t),\\ \mathrm{Pr}(X_{i}\left(t+\Delta t\right)=0|X_{i}\left(t\right)=1) & = & \gamma_{1}\Delta t+o\left(\Delta t\right),\\ \mathrm{Pr}(X_{i}\left(t+\Delta t\right)=1|X_{i}\left(t\right)=1) & = & 1-\gamma_{1}\Delta t-\alpha \Delta t+o\left(\Delta t\right),\\ \mathrm{Pr}(X_{i}\left(t+\Delta t\right)=2|X_{i}\left(t\right)=1) & = & \alpha \Delta t+o(\Delta t),\\ \mathrm{Pr}(X_{i}\left(t+\Delta t\right)=0|X_{i}\left(t\right)=2) & = & \gamma_{2}\Delta t+o\left(\Delta t\right),\\ \mathrm{Pr}(X_{i}\left(t+\Delta t\right)=1|X_{i}\left(t\right)=2) & = & o(\Delta t),\\ \mathrm{Pr}(X_{i}\left(t+\Delta t\right)=2|X_{i}\left(t\right)=2) & = & 1-\gamma_{2}\Delta t+o\left(\Delta t\right). \end{array}$$

Substituting these equations into the above relations and letting Δ*t* → 0, we get the following 3*N*-dimensional differential dynamical system:

$$\begin{array}{c} \left\{ \begin{array}{c} \frac{ds_{i}\left(t\right)}{dt}=\gamma_{1}l_{i}\left(t\right)+\gamma_{2}b_{i}\left(t\right)-\left[\beta_{1}\sum_{j}a_{ij}l_{j}\left(t\right)+\beta_{2}\sum_{j}a_{ij}b_{j}\left(t\right)\right]s_{i}\left(t\right),\;i=1,2,\cdots ,N,\\ \frac{dl_{i}\left(t\right)}{dt}=-\left(\gamma_{1}+\alpha \right)l_{i}\left(t\right)+\left[\beta_{1}\sum_{j}a_{ij}l_{j}\left(t\right)+\beta_{2}\sum_{j}a_{ij}b_{j}\left(t\right)\right]s_{i}\left(t\right),\;i=1,2,\cdots ,N,\\ \frac{db_{i}\left(t\right)}{dt}=\alpha l_{i}\left(t\right)-\gamma_{2}b_{i}\left(t\right),\;i=1,2,\cdots ,N. \end{array}\right. \end{array}$$ (1)

As *s*_*i*_(*t*)+*l*_*i*_(*t*)+*b*_*i*_(*t*) ≡ 1, this system is equal to the following 2*N*-dimensional system:

$$\begin{array}{c} \left\{ \begin{array}{c} \frac{dl_{i}\left(t\right)}{dt}=-\left(\gamma_{1}+\alpha \right)l_{i}\left(t\right)+\left(1-l_{i}\left(t\right)-b_{i}\left(t\right)\right)\left[\beta_{1}\sum_{j}a_{ij}l_{j}\left(t\right)+\beta_{2}\sum_{j}a_{ij}b_{j}\left(t\right)\right],\;\\ \;i=1,2,\cdots ,N,\\ \frac{db_{i}\left(t\right)}{dt}=\alpha l_{i}\left(t\right)-\gamma_{2}b_{i}\left(t\right),\;i=1,2,\cdots ,N. \end{array}\right. \end{array}$$ (2)

We shall refer to system (1) (equivalently, system (2)) as the *node-based SLBS model*.

**Remark 1** *When* *β*_2_ = *γ*_2_ = *α* = 0, *the node-based SLBS model degrades into the* *N*-*interwined SIS model* [27]. *When* *β*_1_ = *β*_2_ *and* *γ*_1_ = *γ*_2_, *our model again degenerates into the* *N*-*interwined SIS model*.

The major task in the subsequent sections is to study the dynamical properties of system (2) (equivalently, system (1)).

## 5 Model analysis

Obviously, system (2) always has the origin as an equilibrium. This trivial equilibrium stands for that all viruses in the network die out almost surely. This section is focused on the stability properties of the trivial equilibrium.

First, consider the asymptotic stability of the trivial equilibrium of system (2). For that purpose, let

$$\begin{array}{c} \Omega =\{x={\left(x_{1},x_{2},\cdots ,x_{2N}\right)}^{T}\in {\mathbb{R}}_{+}^{2N}|x_{i}+x_{i+N}\leq 1,i=1,2,...,N\}. \end{array}$$

Let **x**(*t*) = (*l*_1_(*t*), …, *l*_*N*_(*t*), *b*_1_(*t*), …, *b*_*N*_(*t*))^*T*^, and rewrite system (2) in matrix notation as

$$\begin{array}{c} \frac{dx(t)}{dt}=Bx\left(t\right)+G\left(x\left(t\right)\right) \end{array}$$ (3)

with initial condition **x**(0) ∈ Ω, where

$$\begin{array}{c} B=(\begin{array}{cc} \beta_{1}A-\left(\alpha +\gamma_{1}\right)I & \beta_{2}A\\ \alpha I & -\gamma_{2}I \end{array}), \end{array}$$

$$\begin{array}{ccc} G(x(t)) & = & -(\left(l_{1}\left(t\right)+b_{1}\left(t\right)\right)\left(\beta_{1}\sum_{j}a_{1j}l_{j}\left(t\right)+\beta_{2}\sum_{j}a_{1j}b_{j}\left(t\right)\right),\cdots ,\\ & & {\left(l_{N}\left(t\right)+b_{N}\left(t\right)\right)\left(\beta_{1}\sum_{j}a_{Nj}l_{j}\left(t\right)+\beta_{2}\sum_{j}a_{Nj}b_{j}\left(t\right)\right),0,\cdots ,0)}^{T}. \end{array}$$

Finally, let

$$\begin{array}{c} R_{0}=\frac{\left(\alpha +\gamma_{1}\right)\gamma_{2}}{\beta_{1}\gamma_{2}+\beta_{2}\alpha }. \end{array}$$ (4)

We are ready to present a criterion for the asymptotic stability of the trivial equilibrium.

**Theorem 1** *Consider system (2)*.

*(a)* *The trivial equilibrium is asymptotically stable if* *λ*_max_ < *R*_0_.
*(b)* *The trivial equilibrium is unstable if* *λ*_max_ > *R*_0_.

**Proof** *The characteristic equation for the Jacobian of system (3) evaluated at the trivial equilibrium is*

$$\begin{array}{ccc} \det(\eta I-B) & = & \det(\begin{array}{cc} \left(\eta +\alpha +\gamma_{1}\right)I-\beta_{1}A & -\beta_{2}A\\ -\alpha I & (\eta +\gamma_{2})I \end{array})\\ & = & \det(\left(\eta +\alpha +\gamma_{1}\right)\left(\eta +\gamma_{2}\right)I-\left(\beta_{1}\left(\eta +\gamma_{2}\right)+\beta_{2}\alpha \right)A)\\ & = & 0 \end{array}$$ (5)

*We distinguish between two possibilities*.

*Case 1*: *β*_1_(*γ*_2_ − *γ*_1_) = *α*(*β*_1_ − *β*_2_). *Then*, $R_{0}=\frac{\gamma_{2}}{\beta_{1}}$, *and* Eq (5) *degrades into*

$$\begin{array}{c} \beta_{1}^{N}{\left(\eta +\alpha +\gamma_{1}\right)}^{N}\det\left(\frac{\eta +\gamma_{2}}{\beta_{1}}I-A\right)=0 \end{array}$$

*This equation has* −(*α*+*γ*_1_) *as a root with multiplicity* *N*, *and has* *β*_1_ *λ*_*k*_ − *γ*_2_,1 ≤ *k* ≤ *N* *as the remaining* *N* *roots. If* *λ*_max_ < *R*_0_, *then* *β*_1_ *λ*_*k*_ − *γ*_2_ ≤ *β*_1_ *λ*_max_ − *γ*_2_ < 0 *for all* *k*. *So, the roots of* Eq (5) *are all negative. Hence, the trivial equilibrium of system (2) is asymptotically stable* [38]. *Otherwise, if* *λ*_max_ > *R*_0_, *then* *β*_1_ *λ*_max_ − *γ*_2_ > 0. *So*, Eq (5) *has a positive equilibrium. Thus, the trivial equilibrium is unstable* [38].

*Case 2*: *β*_1_(*γ*_2_ − *γ*_1_) ≠ *α*(*β*_1_ − *β*_2_). *Then*, $-\gamma_{2}-\frac{\alpha \beta_{2}}{\beta_{1}}$ *is not a root of* Eq (5). *Thus*,

$$\begin{array}{c} \det(\frac{\left(\eta +\alpha +\gamma_{1}\right)\left(\eta +\gamma_{2}\right)}{\beta_{1}\left(\eta +\gamma_{2}\right)+\beta_{2}\alpha }I-A)=0. \end{array}$$

*This implies that* *η* *is a root of* Eq (5) *if and only if for some* *k* *(1 ≤ *k* ≤ *N*)*, *η* *is a root of equation*

$$\begin{array}{c} \eta^{2}+a_{k}\eta +b_{k}=0, \end{array}$$ (6)

*where*

$$\begin{array}{ccc} a_{k} & = & \alpha +\gamma_{1}+\gamma_{2}-\beta_{1}\lambda_{k},\\ b_{k} & = & \left(\alpha +\gamma_{1}\right)\gamma_{2}-\lambda_{k}\left(\beta_{1}\gamma_{2}+\beta_{2}\alpha \right). \end{array}$$

*If* *λ*_max_ < *R*_0_, *we have* *a*_*k*_ > 0 and *b*_*k*_ > 0. *So, it follows from the Hurwitz criterion* [38] *that the two roots of* Eq (6) *have negative real parts. As a result, all roots of* Eq (5) *have negative real parts. Hence, the trivial equilibrium is asymptotically stable* [38]. *Otherwise, if* *λ*_max_ > *R*_0_, *the equation*

$$\begin{array}{c} \eta^{2}+a_{1}\eta +b_{1}=0 \end{array}$$

*has a root with positive real part. As a result*, Eq (5) *has a root with positive real part. Hence, the trivial equilibrium is unstable* [38]. *The proof is complete*.

**Remark 2** *This theorem can also be formulated as (a)* *λ*_max_ < *R*_0_ ⇒ *s*(**B**) < 0, *and (b)* *λ*_max_ > *R*_0_ ⇒ *s*(**B**) > 0.

Second, consider the global stability of the trivial equilibrium of system (2). For that purpose, the following lemma is indispensable.

**Lemma 4** *The set Ω is positively invariant for system (2). That is*, **x**(0) ∈ Ω *implies* **x**(*t*) ∈ Ω *for all* *t* > 0.

**Proof** ∂Ω *consists of the following* 3*N* *hyperplanes*:

$$\begin{array}{c} S_{i}=\left\{x\in \Omega |x_{i}=0\right\},\;1\leq i\leq N, \end{array}$$

$$\begin{array}{c} T_{i}=\left\{x\in \Omega |x_{i+N}=0\right\},\;1\leq i\leq N, \end{array}$$

$$\begin{array}{c} U_{i}=\left\{x\in \Omega |x_{i}+x_{i+N}=1\right\},\;1\leq i\leq N. \end{array}$$

*For* 1 ≤ *i* ≤ *N*, *S*_*i*_, *T*_*i*_, *and* *U*_*i*_ *have*

$$\begin{array}{c} n_{i}=\left(0,\cdots ,0,\underset{i}{\underset{︸}{-1}},0,\cdots ,0\right), \end{array}$$

$$\begin{array}{c} n_{N+i}=\left(0,\cdots ,0,\underset{N+i}{\underset{︸}{-1}},0,\cdots ,0\right), \end{array}$$

*and*

$$\begin{array}{c} n_{2N+i}=\left(0,\cdots ,0,\underset{i}{\underset{︸}{1}},0,\cdots ,0,\underset{N+i}{\underset{︸}{1}},0,\cdots ,0\right)) \end{array}$$

*as their respective outer normal vectors. Let* **x** *be a smooth point of* ∂Ω. *We distinguish among three possibilities*.

*Case 1*: *x*_*i*_ = 0 *for some* 1 ≤ *i* ≤ *N*. *Then*, *x*_*N*+*i*_ < 1, *and* *x*_*j*_ > 0 *for all* *j* ≠ *i*. *As graph* *G* *has no isolated node, we have*

$$\begin{array}{c} \left\langle \text{Bx}+G\left(x\right),n_{i}\right\rangle =-\left(1-x_{N+i}\right)\left[\beta_{1}\sum_{j}a_{ij}x_{j}+\beta_{2}\sum_{j}a_{ij}x_{N+j}\right]<0. \end{array}$$

*Case 2*: *x*_*N*+*i*_ = 0 *for some* 1 ≤ *i* ≤ *N*. *Then*, *x*_*i*_ > 0. *Thus*,

$$\begin{array}{c} \left\langle \text{Bx}+G\left(x\right),n_{N+i}\right\rangle =-\alpha x_{i}<0. \end{array}$$

*Case 3*: *x*_*i*_ + *x*_*N* + *i*_ = 1 *for some* 1 ≤ *i* ≤ *N*. *Then*,

$$\begin{array}{c} \left\langle \text{Bx}+G\left(x\right),n_{2N+i}\right\rangle =-\gamma_{1}x_{i}-\gamma_{2}\left(1-x_{i}\right)<0. \end{array}$$

*Combining the above discussions, we get that* **Bx** + **G**(**x**) *is pointing into* ∂Ω. *The claimed result then follows from Lemma 1. The proof is complete*.

We are ready to present a criterion for the global stability of the trivial equilibrium.

**Theorem 2** *The trivial equilibrium of system (2) is globally asymptotically stable if* *λ*_max_ < *R*_0_.

**Proof** *Look at system (3). As matrix* **B** ^*T*^ *is irreducible and its off-diagonal entries are all non-negative, it follows from* [36] *that* **B** ^*T*^ *has a positive eigenvector* **z** = (*z*_1_, *z*_2_, ⋯, *z*_2*N*_) *belonging to its eigenvalue* *s*(**B** ^*T*^). *Let* *r* = min_*i*_ *z*_*i*_ (> 0). *Then, for all* **x** ∈ Ω, *we have*

$$\begin{array}{ccc} \left\langle x,z\right\rangle & \geq & r\sum_{i}x_{i}=r{∥x∥}_{1},\\ \left\langle G(x),z\right\rangle & = & -\sum_{i}z_{i}\left(x_{i}+x_{N+i}\right)\left(\beta_{1}\sum_{j}a_{ij}x_{j}+\beta_{2}\sum_{j}a_{ij}x_{j+N}\right)\leq 0 \end{array}$$

*Moreover*, ⟨**G**(**x**), **z**⟩ = 0 *implies that* **x** = **0**. *In view of Theorem 1 and Lemma 3, the claimed result follows from Lemma 2. The proof is complete*.

**Remark 3** *The global stability of the trivial equilibrium of system (2) implies that, almost surely, the viruses in the network decline toward extinction*.

Next, consider the global exponential stability of the trivial equilibrium of system. For that purpose, let

$$\begin{array}{c} R_{1}=\frac{\gamma_{1}}{\beta_{1}}. \end{array}$$ (7)

Now, let us give a criterion for the global exponential stability of the trivial equilibrium.

**Theorem 3** *The trivial equilibrium of system (2) is globally exponentially stable if* *λ*_max_ < *R*_1_.

**Proof** *We have*

$$\begin{array}{ccc} \frac{d(l_{i}\left(t\right)+b_{i}\left(t\right))}{dt} & = & \beta_{1}\left(1-l_{i}\left(t\right)-b_{i}\left(t\right)\right)\sum_{j}a_{ij}\left(l_{j}\left(t\right)+b_{j}\left(t\right)\right)-\gamma_{1}\left(l_{i}\left(t\right)+b_{i}\left(t\right)\right)\\ & & -\left(\beta_{1}-\beta_{2}\right)\left(1-l_{i}\left(t\right)-b_{i}\left(t\right)\right)\sum_{j}a_{ij}b_{j}\left(t\right)-\left(\gamma_{2}-\gamma_{1}\right)b_{i}\left(t\right)\\ & \leq & \beta_{1}\left(1-l_{i}\left(t\right)-b_{i}\left(t\right)\right)\sum_{j}a_{ij}\left(l_{j}\left(t\right)+b_{j}\left(t\right)\right)-\gamma_{1}\left(l_{i}\left(t\right)+b_{i}\left(t\right)\right)\\ & \leq & \beta_{1}\sum_{j}a_{ij}\left(l_{j}\left(t\right)+b_{j}\left(t\right)\right)-\gamma_{1}\left(l_{i}\left(t\right)+b_{i}\left(t\right)\right),\;i=1,\cdots ,N. \end{array}$$

*Consider the comparison system*

$$\begin{array}{c} \frac{dp_{i}\left(t\right)}{dt}=\beta_{1}\sum_{j}a_{ij}p_{j}\left(t\right)-\gamma_{1}p_{i}\left(t\right),\;i=1,\cdots ,N, \end{array}$$

*with initial condition* *p*_*i*_(0) = *l*_*i*_(0)+*b*_*i*_(0), *i* = 1, ⋯, *N*. Let **p**(*t*) = [*p*_1_(*t*), ⋯, *p*_*N*_(*t*)]^*T*^ *and rewrite the comparison system in matrix notation as*

$$\begin{array}{c} \frac{dp(t)}{dt}=\left(\beta_{1}A-\gamma_{1}I\right)p\left(t\right). \end{array}$$

*The solution to this comparison system is*

$$\begin{array}{c} p\left(t\right)=e^{(\beta_{1}A-\gamma_{1}I)t}p\left(0\right) \end{array}$$

*Let* **U** *denote the* *N* × *N* *matrix having an eigenvector belonging to the eigenvalue* *λ*_*i*_ *of matrix* **A** *as the* *i*-*th column. Then, we have the following spectral decomposition of matrix* **A**:

$$\begin{array}{c} A=U\times diag\left(\lambda_{1},\lambda_{2},...,\lambda_{N}\right)\times U^{T}. \end{array}$$

*As a result, we have*

$$\begin{array}{c} p\left(t\right)=U\times diag\left(e^{(\beta_{1}\lambda_{1}-\gamma_{1})t},e^{(\beta_{1}\lambda_{2}-\gamma_{1})t},...,e^{(\beta_{1}\lambda_{N}-\gamma_{1})t}\right)\times U^{T}\times p\left(0\right) \end{array}$$

*Hence*,

$$\begin{array}{ccc} {∥p\left(t\right)∥}_{1} & \leq & {∥U∥}_{1}\times ∥diag\left(e^{(\beta_{1}\lambda_{1}-\gamma_{1})t},e^{(\beta_{1}\lambda_{2}-\gamma_{1})t},...,e^{(\beta_{1}\lambda_{N}-\gamma_{1})t}\right){∥}_{1}\times ∥U^{T}{∥}_{1}\times {∥p\left(0\right)∥}_{1}\\ & = & e^{(\beta_{1}\lambda_{\max}-\gamma_{1})t}\times {∥U∥}_{1}\times ∥U^{T}{∥}_{1}\times {∥p\left(0\right)∥}_{1}. \end{array}$$

*Therefore*, **p**(*t*) → **0** *at an exponential speed if* *λ*_max_ < *R*_1_. *It follows from the comparison theorem* [38] *that if* *λ*_max_ < *R*_1_, *then for each* *i*, *l*_*i*_(*t*) + *b*_*i*_(*t*) → 0 *at an exponential speed. The proof is complete*.

Finally, let us consider what happens if *λ*_max_ > *R*_0_. By applying Lemma 2 to system (2) and in view of Theorem 1, we get the following result.

**Theorem 4** *Consider system (2). If* *λ*_max_ > *R*_0_, *then*

$$\begin{array}{c} \sum_{i}\left(l_{i}\left(0\right)+b_{i}\left(0\right)\right)>0\Rightarrow \underset{t\to \infty }{lim inf}\sum_{i}\left(l_{i}\left(t\right)+b_{i}\left(t\right)\right)>0. \end{array}$$

**Remark 4** *This theorem shows that if* *λ*_max_ > *R*_0_, *then, almost surely, viruses in the network persist*.

**Remark 5** *As the largest eigenvalue of a network is an indicator of the structure of the network, Theorems 1–4 clearly reveal the impact of the network topology on the viral prevalence; a network with smaller largest eigenvalue is inclined to contain viruses*.

It follows from Theorems 1–4 that it is proper to partition the value range (0, ∞) of *λ*_max_ into three subintervals: *I*_1_ = (0, *R*_1_), *I*_2_ = (*R*_1_, *R*_0_), and *I*_3_ = (*R*_0_, ∞). When *λ*_max_ ∈ *I*_1_, viruses in the network tends to extinction almost surely, at an exponential speed. When *λ*_max_ ∈ *I*_2_, viruses in the network declines toward annihilation almost surely. When *λ*_max_ ∈ *I*_3_, viruses in the network persist.

## 6 Numerical examples

In this section, we shall verify the results obtained in the previous section using numerical examples. For that purpose, let *p*(*t*) denote the percentage of infected nodes in all nodes at time *t*, $p(t)=\frac{1}{N}\sum_{i}(l_{i}(t)+b_{i}(t))$.

**Example 1** *Consider the node-based SLBS model, and take a complete graph on 100 nodes as the viral propagation network. Then*, *λ*_max_ = 99.

**Fig 2:**
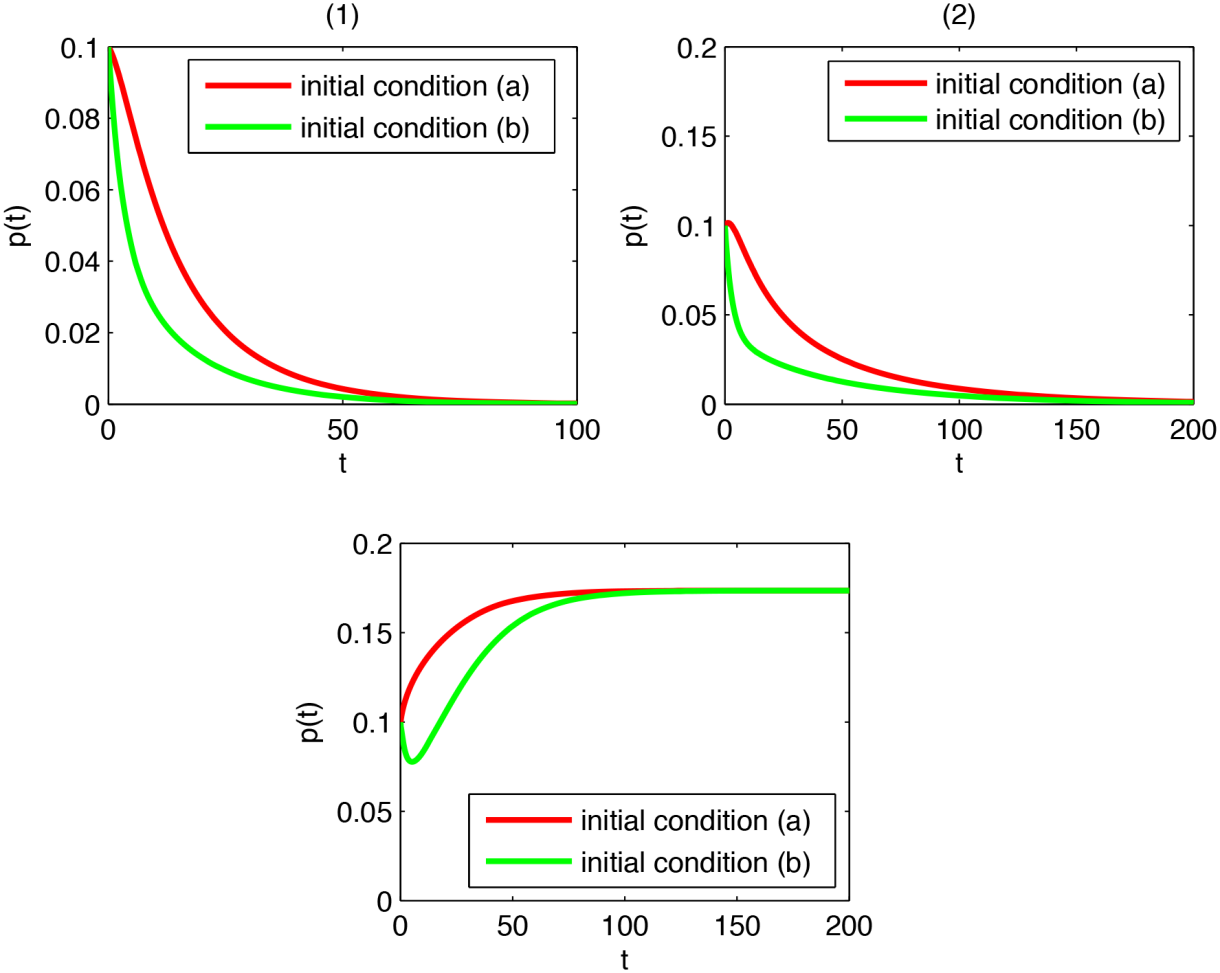
(1) *p*(*t*) in the case *λ*_max_ ∈ *I*_1_; (2) *p*(*t*) in the case *λ*_max_ ∈ *I*_2_; (3) *p*(*t*) in the case *λ*_max_ ∈ *I*_3_.

1. *Suppose* *β*_1_ = 0.002, *β*_2_ = 0.001, *γ*_1_ = 0.2, *γ*_2_ = 0.3, *and* *α* = 0.1. *As* *λ*_max_ ∈ *I*_1_, *Theorem 3 predicts that* *p*(*t*) → 0 *at an exponential speed*. Fig 2(1) *shows the trend of* *p*(*t*) *provided (a) there are initially 90 susceptible nodes and 10 latent nodes, or (b) there are initially 90 susceptible nodes and 10 exploding nodes. It can be seen that viruses tend to extinction very quickly, in consistency with the prediction*.
2. *Suppose* *β*_1_ = 0.0025, *β*_2_ = 0.001, *γ*_1_ = 0.2, *γ*_2_ = 0.3, *and* *α* = 0.1. *As* *λ*_max_ ∈ *I*_2_, *Theorem 2 predicts that* *p*(*t*) → 0. Fig 2(2) *shows the trend of* *p*(*t*) *provided (a) there are initially 90 susceptible nodes and 10 latent nodes, or (b) there are initially 90 susceptible nodes and 10 exploding nodes. It can be seen that viruses tend toward annihilation, in agreement with the prediction*.
3. *Suppose* *β*_1_ = 0.01, *β*_2_ = 0.005, *γ*_1_ = 0.2, *γ*_2_ = 0.3, *and* *α* = 0.1. *As* *λ*_max_ ∈ *I*_3_, *Theorem 4 predicts that* *p*(*t*) ↛ 0. Fig 2(3) *shows the trend of* *p*(*t*) *provided (a) there are initially 90 susceptible nodes and 10 latent nodes, or (b) there are initially 90 susceptible nodes and 10 exploding nodes. It can be seen that viruses persist, agreeing with the prediction*.

**Example 2** *Consider the node-based SLBS model, and take a star-shaped graph on 100 nodes as the viral propagation network. Then*, $\lambda_{\text{max}}=\sqrt{99}=9.95$.

**Fig 3:**
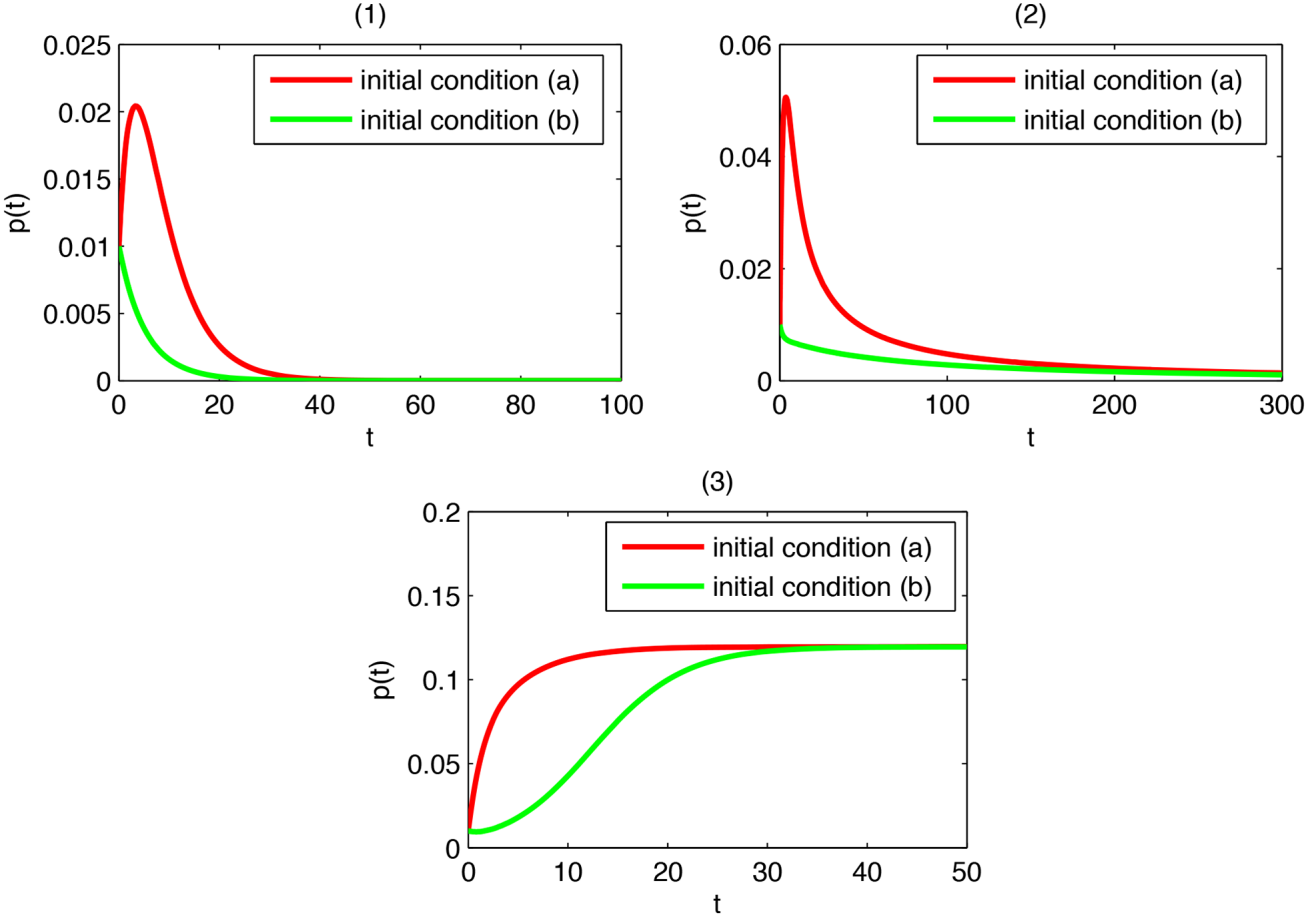
(1) *p*(*t*) in the case *λ*_max_ ∈ *I*_1_; (2) *p*(*t*) in the case *λ*_max_ ∈ *I*_2_; (3) *p*(*t*) in the case *λ*_max_ ∈ *I*_3_.

1. *Suppose* *β*_1_ = 0.01, *β*_2_ = 0.006, *γ*_1_ = 0.2, *γ*_2_ = 0.3, *and* *α* = 0.1. *As* *λ*_max_ ∈ *I*_1_, *Theorem 3 predicts that* *p*(*t*) → 0 *at an exponential speed*. Fig 3(1) *shows the trend of* *p*(*t*) *provided (a) the hub is initially latent, and the remaining 99 nodes are initially susceptible, or (b) one leaf node is initially latent, and the remaining 99 nodes are initially susceptible. It can be seen that viruses tend to extinction very quickly, coinciding with the prediction*.
2. *Suppose* *β*_1_ = 0.035, *β*_2_ = 0.01, *γ*_1_ = 0.2, *γ*_2_ = 0.4, *and* *α* = 0.2. *As* *λ*_max_ ∈ *I*_2_, *Theorem 2 predicts that* *p*(*t*) → 0. Fig 3(2) *shows the trend of* *p*(*t*) *provided (a) the hub is initially latent, and the remaining 99 nodes are initially susceptible, or (b) one leaf node is initially latent, and the remaining 99 nodes are initially susceptible. It can be seen that viruses tend to extinction, in agreement with the prediction*.
3. *Suppose* *β*_1_ = 0.05, *β*_2_ = 0.02, *γ*_1_ = 0.2, *γ*_2_ = 0.3, *and* *α* = 0.1. *As* *λ*_max_ ∈ *I*_3_, *Theorem 4 predicts that* *p*(*t*) ↛ 0. Fig 3(3) *shows the trend of* *p*(*t*) *provided (a) the hub is initially latent, and the remaining 99 nodes are initially susceptible, or (b) one leaf node is initially latent, and the remaining 99 nodes are initially susceptible. It can be seen that viruses persist, consistent with the prediction*.

**Example 3** *Consider the node-based SLBS model, and take an Erdos-Renyi graph on 50 nodes, which is produced randomly with connection probability 0.2, as the viral propagation network. Numerical calculation gives* *λ*_max_ = 10.19.

**Fig 4:**
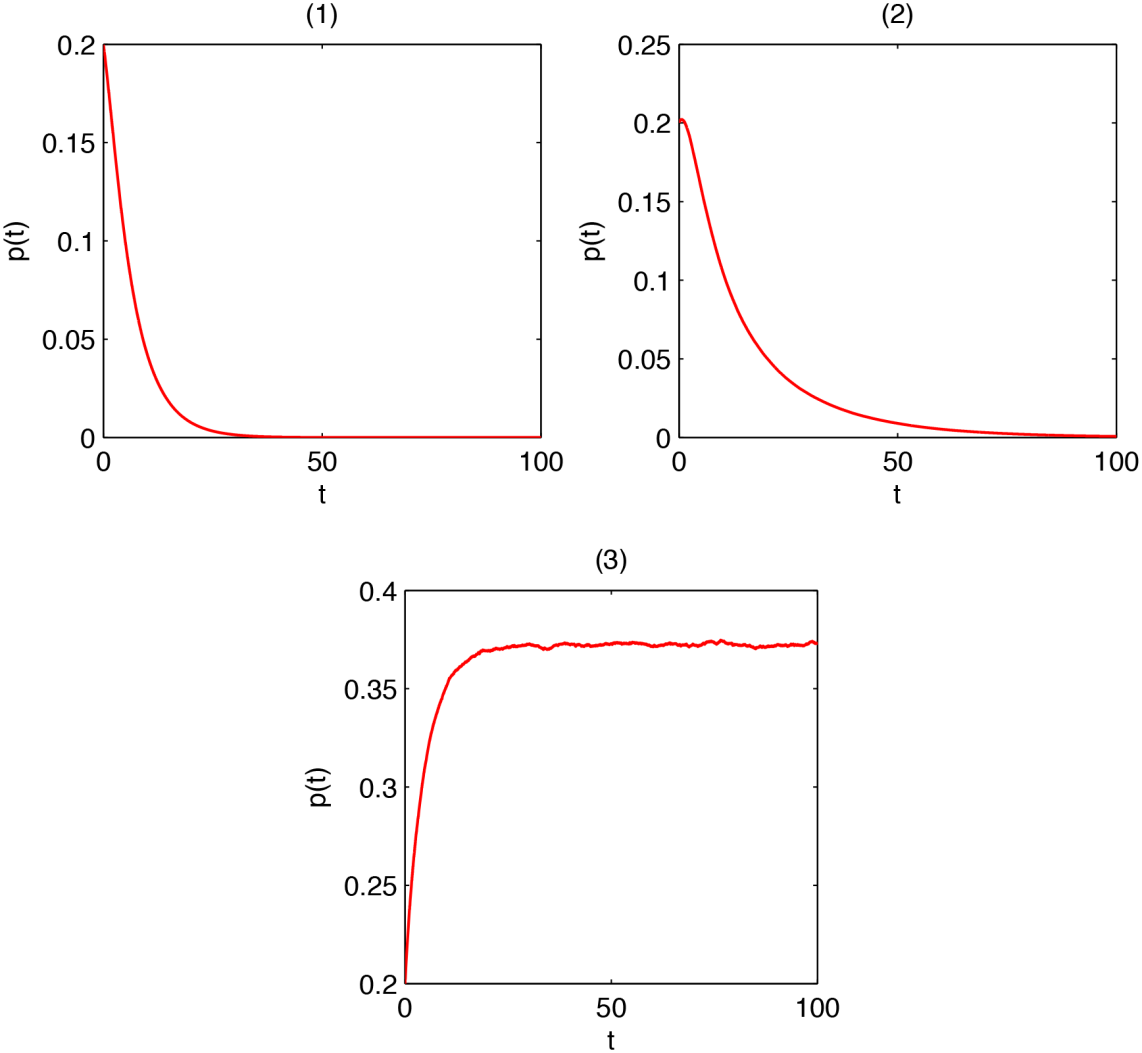
(1) *p*(*t*) in the case *λ*_max_ ∈ *I*_1_; (2) *p*(*t*) in the case *λ*_max_ ∈ *I*_2_; (3) *p*(*t*) in the case *λ*_max_ ∈ *I*_3_.

1. *Suppose* *β*_1_ = 0.015, *β*_2_ = 0.01, *γ*_1_ = 0.2, *γ*_2_ = 0.4, *and* *α* = 0.2. *As* *λ*_max_ ∈ *I*_1_, *Theorem 3 predicts that* *p*(*t*) → 0 *at an exponential speed*. Fig 4(1) *shows the trend of* *p*(*t*) *provided there are initially 10 latent nodes and 40 susceptible nodes. It can be seen that viruses go to extinction very quickly, in consistency with the prediction*.
2. *Suppose* *β*_1_ = 0.03, *β*_2_ = 0.01, *γ*_1_ = 0.2, *γ*_2_ = 0.4, *and* *α* = 0.2. *As* *λ*_max_ ∈ *I*_2_, *Theorem 2 predicts that* *p*(*t*) → 0. Fig 4(2) *shows the trend of* *p*(*t*) *provided there are 10 latent nodes and 40 susceptible nodes. It can be seen that viruses tend toward annihilation, in coherence with the prediction*.
3. *Suppose* *β*_1_ = 0.05, *β*_2_ = 0.03, *γ*_1_ = 0.2, *γ*_2_ = 0.4, *and* *α* = 0.2. *As* *λ*_max_ ∈ *I*_3_, *Theorem 4 predicts that* *p*(*t*) ↛ 0. Fig 4(3) *shows the trend of* *p*(*t*) *provided there are initially 10 latent nodes and 40 susceptible nodes. It can be seen that viruses persist, in agreement with the prediction*.

In a word, the above given numerical examples are all in perfect agreement with the theoretical results.

## 7 Further discussions

It can be seen from the main results in Section 5 that an effective approach to the containment of electronic virus is to adjust the system parameters so that *R*_0_ or *R*_1_ is large enough. Simple calculations yield

$$\begin{array}{c} \frac{\partial R_{0}}{\partial \beta_{1}}<0,\;\frac{\partial R_{0}}{\partial \beta_{2}}<0,\;\frac{\partial R_{0}}{\partial \gamma_{1}}>0,\;\frac{\partial R_{0}}{\partial \gamma_{2}}>0,\;\frac{\partial R_{1}}{\partial \alpha }>0,\\ \frac{\partial R_{1}}{\partial \beta_{1}}<0,\;\frac{\partial R_{0}}{\partial \gamma_{1}}>0, \end{array}$$

As a result, the following practical measures are strongly recommended.

- Install and timely update antivirus software on computers, so as to reduce the two cure rates of infected computers.
- Filter and block suspicious messages with firewall located at the gateway of a domain, so as to lower the two infecting rates of susceptible computers.

On the other hand, it benefits the inhibition of virus to adjust the structure of the propagation network so that its maximum eigenvalue is small enough. As there is no closed-form formula for the maximum eigenvalue of a general adjacency matrix, it is difficult to verify this condition. To circumvent this difficulty, let us present an easily verified condition for the final extinction of virus as follows.

**Theorem 5** All viruses in a network would tend to extinction if

$$\begin{array}{c} \min\{\sqrt{\frac{N-1}{N}\sum_{i}d_{i}},\max_{i}d_{i}\}<\max\left\{R_{0},R_{1}\right\}. \end{array}$$

**Proof** *The claim follows by combining Lemma 3 and Theorems 2–3*.

This theorem suggests that simultaneously reducing the number of links and the maximum node degree in a network should contribute to the annihilation of virus.

## 8 Conclusions and remarks

To understand the way that the spread of virus on a network is affected by the structure of the network, a new epidemic model of computer virus has been proposed. The model analysis reveals that the maximum eigenvalue of the network is a key factor determining the viral prevalence; viruses tend to extinction very quickly or approach extinction or persist depending on where the maximum eigenvalue of the network lies. As a result, viruses can be contained by properly adjusting the structure of the propagation network.

Towards this direction, lots of work has yet to be done. For instance, our model assumes that all computers have the same infection rate, the same bursting rate, and the same curing rate. In reality, however, these rates vary from computer to computer. Hence, our model should be generalized so that different nodes have different infection rates, different bursting rates, and different curing rates. Additionally, that computers are likely to be infected by removable storage media [39] may lead to the emergence of a non-trivial steady state. In this situation, it makes sense to suppress the fraction of the infected nodes. Third, the immunization strategy we adopt also has significant impact on the viral prevalence. To a certain extent, the static immunization problem reduces to that of assigning different curing rates to different nodes so that the best virus containment effect is achieved, given that the sum of curing rates of all nodes is fixed [33, 40], while the dynamic immunization problem can be solved by use of the optimal control theory [41]. Last, but not least, the methodology developed in this work can be applied to the situation of infectious diseases [42–45].
