# Correction: The Impact of the Network Topology on the Viral Prevalence: A Node-Based Approach

**DOI:** 10.1371/journal.pone.0137849

**Published:** 2015-09-03

**Authors:** The PLOS ONE Staff

There is an error in Fig 1 Please see the correct Fig 1 here.

**Fig 1:**
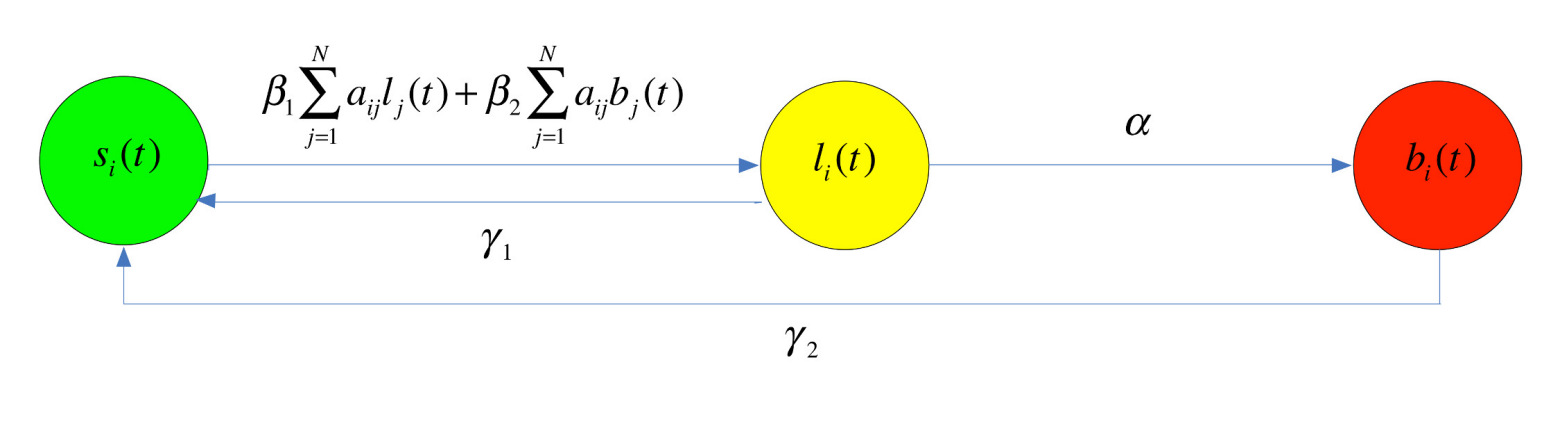
Diagram of assumptions (H1)-(H3).
